# Assay of phospholipases A_2_ and their inhibitors by kinetic analysis in the scooting mode

**DOI:** 10.1155/S0962935192000164

**Published:** 1992

**Authors:** Mahendra Kumar Jain, Bao-Zhu Yu, Michael H. Gelb, Otto G. Berg

**Affiliations:** 1 Department of Chemistry and Biochemistry University of Delaware Newark DE 19716 USA; 2 Departments of Chemistry and Biochemistry University of Washington Seattle WA 98195 USA; 3 Department of Molecular Biology Uppsala University Biomedical Center Uppsala Sweden

## Abstract

Several cellular processes are regulated by interfacial catalysis on biomembrane surfaces. Phospholipases A_2_ (PLA_2_) are interesting not only as prototypes for interfacial catalysis, but also because they mobilize precursors for the biosynthesis of eicosanoids and platelet activating factor, and these agents ultimately control a wide range of secretory and inflammatory processes. Since PLA_2_ carry out their catalytic function at membrane surfaces, the kinetics of these enzymes depends on what the enzyme ‘sees’ at the interface, and thus the observed rate is profoundly influenced by the organization and dynamics of the lipidwater interface (‘quality of the interface’). In this review we elaborate the advantages of monitoring interfacial catalysis in the scooting mode, that is, under the conditions where the enzyme remains bound to vesicles for several thousand catalytic turnover cycles. Such a highly processive catalytic turnover in the scooting mode is useful for a rigorous and quantitative characterization of the kinetics of interfacial catalysis. This analysis is now extended to provide insights into designing strategy for PLA_2_ assays and screens for their inhibitors.


Mediators of Inflammation, 1, 85–100 (1992)

### Assay of phospholipases $A_2$ and their inhibitors by kinetic analysis in the scooting mode

Mahendra Kumar Jain[1,CA], Bao-Zhu Yu[1], Michael H. Gelb[2] and Otto G. Berg[3]

[1] Department of Chemistry and Biochemistry, University of Delaware, Newark, DE 19716, USA;
[2] Departments of Chemistry and Biochemistry, University of Washington, Seattle, WA 98195, USA;
[3] Department of Molecular Biology, Uppsala University Biomedical Center, Uppsala, Sweden

[CA] Corresponding Author

**SEVERAL cellular processes are regulated by interfacial catalysis on biomembrane surfaces. Phospholipases $A_2$ ($PLA_2$) are interesting not only as prototypes for interfacial catalysis, but also because they mobilize precursors for the biosynthesis of eicosanoids and platelet activating factor, and these agents ultimately control a wide range of secretory and inflammatory processes. Since $PLA_2$ carry out their catalytic function at membrane surfaces, the kinetics of these enzymes depends on what the enzyme 'sees' at the interface, and thus the observed rate is profoundly influenced by the organization and dynamics of the lipid–water interface ('quality of the interface'). In this review we elaborate the advantages of monitoring interfacial catalysis in the scooting mode, that is, under the conditions where the enzyme remains bound to vesicles for several thousand catalytic turnover cycles. Such a highly processive catalytic turnover in the scooting mode is useful for a rigorous and quantitative characterization of the kinetics of interfacial catalysis. This analysis is now extended to provide insights into designing strategy for $PLA_2$ assays and screens for their inhibitors.**

**Key words:** Catalysis in the scooting mode, Competitive inhibitors, Interfacial catalysis, Neutral diluent, Phospholipase $A_2$

### Introduction

Interfacial catalysis is an intriguing biophysical phenomenon because it involves a complex interplay of physical and biochemical processes. Recently it has attracted considerable attention as such processes are implicated in signal transduction and other cellular regulatory mechanisms in which water-soluble proteins are forced to act on the substrate localized in biomembranes. Several classes of proteins are known to be functionally active at interfaces; for example, lipolytic enzymes, protein and acylglycerol kinases, acyltransferases and certain glycosidases. Interfacial catalysis by lipolytic enzymes is involved in diverse processes such as the digestion of fats, the tailoring of membrane lipids, digestion of phagocytosed bacteria, and the modulation of signal transduction.[1,2] A resurgence of interest in $PLA_2$ has been sparked by the possibility that the release of arachidonate and lysophospholipids from membrane phospholipids is probably the rate-limiting step in the biosynthesis of eicosanoids (prostaglandins, thromboxanes, leukotrienes, lipoxins, hydroxyeicosatetraenoic acids) and may be involved in the generation of the precursor for platelet activating factor. These regulatory molecules ultimately control a wide range of physiological and pathological processes leading to inflammation, rheumatoid arthritis, asthma, ischaemia, toxic shock, psoriasis, pancreatitis, and burn trauma.

### Molecular characteristics of phospholipases $A_2$

By definition, $PLA_2$ hydrolyse the *sn*-2-ester bond in 1,2-diacylglycero-*sn*-3-phospholipids. Two classes of soluble $PLA_2$ have been adequately characterized, secretory and cytoplasmic $PLA_2$.

*Secretory $PLA_2$:* Secretory phospholipases $A_2$ are ubiquitous in secretory granules of inflammatory cells and in extracellular exudates (pancreatic, ascitis, synovial and peritoneal), and venoms (snakes, bees, or lizards). Their molecular and catalytic properties are similar, which suggests a

**Abbreviations:** DMPC, dimyristoyl glycero-*sn*-3-phosphocholine; DMPM, dimyristoyl glycero-*sn*-3-phosphomethanol; DTPC, ditetradecyl glycero-*sn*-3-phosphocholine; DTPM, ditetradecyl glycero-*sn*-3-phosphomethanol; HDNS, dansyl-hexadecylphosphoethanolamine; 2H-GPC, 2-hexadecyl-glycero-*sn*-3-phosphocholine; NK-529, 1,3,3,1′,3′,3′-hexamethyl-indocarbocyananine; $PLA_2$, phospholipases $A_2$ from pig pancreas unless stated otherwise; proPLA, prophospholipases $A_2$ from pig pancreas.

© 1992 Rapid Communications of Oxford Ltd role for these enzymes in digestion and defence mechanisms, including macrophage functions. The genes coding for type I $PLA_2$ (in pancreas, spleen, gastric mucosa, lung, stomach, kidney, and elapid snakes of the Old World) and type II $PLA_2$ (in venoms of crotalids and viperids of the New World, platelets, macrophages, ascites, neutrophils, placenta, tonsil, kidney, synovial fluid and liver mitochondria) are localized on chromosomes 12 and 1, respectively.[3] These stable and water-soluble enzymes contain about 120 amino acids in a highly conserved single polypeptide chain with a rigid three-dimensional structure stabilized by seven disulphide bridges.[4,5]

The two types of $PLA_2$ are distinguished by the translocation of Cys from positions 11 and 77 in type I, to 50 and 132 in type II, which also contains seven additional amino acid residues at the C-terminus. Calcium is an essential cofactor for the binding of the substrate to the active site via Asp-49 and a highly conserved loop. The $K_{Ca}$ values for these enzymes are in the 0.1 to 3 mM range. The invariant catalytic triad consists of His-48, Asp-99 and a water molecule. Secretory $PLA_2$ have relatively broad substrate specificities and apparent catalytic turnover numbers of 50 to 1 000 $s^{-1}$. Structural analysis by X-ray diffraction[6-9] has revealed a common three-dimensional architecture in which five of the seven disulphide bridges are conserved in $PLA_2$ of types I and II, and these structures are not noticeably altered by the binding of substrate analogues to the active site. Although the bee venom phospholipase $A_2$ (type III) has a considerably different architecture, the conformations of all the three types of $PLA_2$ in the region of the substrate binding site are essentially the same and involve similar amino acid residues.

*Cytoplasmic $PLA_2$:* Cytoplasmic phospholipases $A_2$ of higher molecular weight (40–110 kDa range) have been reported in the cytoplasm of several cell types. An 86 kDa protein has been cloned[10,11] and the cDNA inferred amino acid sequence shows no homology with that of the secretory $PLA_2$. Also, calcium and other divalent ions promote binding of cytoplasmic $PLA_2$ to the substrate interface through a specific domain.[10-13] The properties and substrate specificity of these cytoplasmic $PLA_2$ suggest that they may be primarily involved in the mobilization of arachidonate triggered by submicromolar concentrations of calcium required for the binding of the enzyme to the interface.

Phospholipase $A_2$ activities with very different characteristics have been reported in a variety of tissues, and their relationships to these two classes of $PLA_2$ is not yet established. Similarly, the role and regulation of $PLA_2$ in the inflammatory processes is not clear. Secretory $PLA_2$ accumulate in certain body fluids during inflammatory responses; however, it is not certain whether phospholipase $A_2$ causes inflammation or is produced in response to inflammation. In this review we focus on the interfacial kinetic properties of secretory phospholipases $A_2$, primarily because they are well characterized. However, the kinetic behavior of cytoplasmic $PLA_2$ abides by the same general principles of interfacial catalysis.[13]

### The kinetic paradigm

Key features of interfacial catalysis are adequately represented by the minimal kinetic scheme shown in Fig. 1. The theoretical basis for this scheme[14,15] and its broad implications[15-20] are elaborated elsewhere. An appreciation of this scheme is crucial for understanding the problems encountered in commonly used assays for activity and inhibition of $PLA_2$.

*Catalysis occurs at the interface:* In interfacial catalysis it is important to consider what the enzyme 'sees' under a given set of experimental conditions, rather than only considering what is present in the reaction mixture. Although $PLA_2$ are water soluble, they have evolved to carry out catalysis at the interface, because in aqueous dispersions phospholipids spontaneously organize into aggregates.[21] The magnitude of the hydrophobic effect is so large that the concentration of solitary, monomeric, naturally occurring phospholipids in the aqueous phase is very low (<100 pM). Although $PLA_2$ obey the Michaelis–Menten formalism, their observed kinetic behaviour differs from the classical kinetics of a soluble enzyme acting on solitary monomeric substrate in solution[22] in two major ways:[4,14,23]

(a) The catalytic turnover of solitary monomers of short chain zwitterionic phospholipids

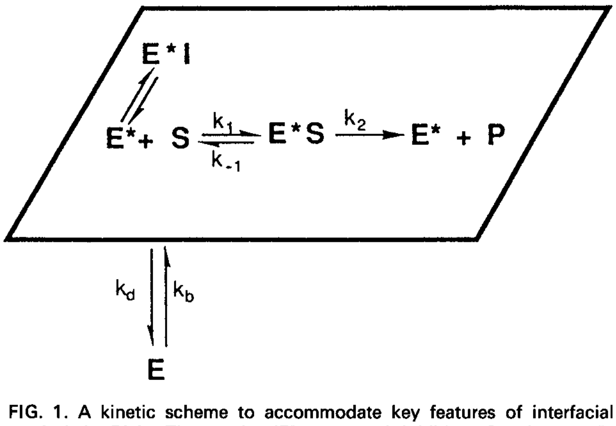

FIG. 1. A kinetic scheme to accommodate key features of interfacial catalysis by $PLA_2$. The species (E*, enzyme; I, inhibitor; S, substrate; P, products) shown in the box plane are in or bound to the bilayer, and the enzyme in the aqueous phase is shown as E. A similar scheme can be adopted for other lipid–water interfaces, however, in such cases the exchange of the substrate and products must be explicitly considered.

is often considerably slower than that on the aqueous dispersions of long chain substrates under optimum conditions.

(b) The composition, dispersity, organization and dynamics of all the components at the interface determine the overall effective rate of catalytic turnover. Such factors determine not only the proportion of the enzyme at the interface but also the local concentration (mole fraction) of the substrate and products that the enzyme 'sees' during the course of catalysis at the interface.

Within the paradigm of the kinetic scheme in Fig. 1, phospholipase $A_2$ is distributed between the aqueous phase and the interface, and catalysis is mediated only by the enzyme at the interface (designated as E*). Thus the concentration of the substrate is important in two ways:[14,15] the bulk concentration, most conveniently expressed in units of moles/litre, controls the fraction of the enzyme at the interface. On the other hand, the mole fraction of the substrate in the interface determines the probability of the encounter of the substrate with the enzyme at the interface, that is, it is the concentration that the bound enzyme 'sees' for the formation of the Michaelis–Menten complex (E*S). Thus the effective rate of hydrolysis is determined not only by the number density of substrate molecules in the interface as related to the mole fraction of the substrate, but also by the E to E* equilibrium that depends upon the bulk concentration of the interface.

*The apparent catalytic turnover is influenced by the interface:* The behaviour of amphipathic molecules in aqueous dispersions and factors governing the organization and dynamics of phospholipids at the interface are now reasonably well understood.[21–24] The hydrophobic effect provides the driving force for amphipathic molecules in water to form organized structures, such as bilayers, monolayers, micelles and emulsions. In such aggregates, the dynamics of exchange of monomers depends on the shape of the amphiphile, and on the balance between the hydrophobic and hydrophilic forces on the amphiphile in the aqueous environment. The free energy for the removal of long chain phospholipid molecules from a bilayer to the surrounding aqueous phase exceeds 20 kcal/mole. Therefore, the concentration of solitary monomers in the aqueous phase is well below 100 pM. Under such conditions intervesicle exchange of phospholipids has a half-time of several hours. Amphiphiles in micelles undergo faster exchange because of fission and fusion of micelles.

The enzyme at the interface also facilitates the steps involved in the catalytic turnover.[25,26] In the context of the scheme shown in Fig. 1, the apparent activation of $PLA_2$ at the interface is a necessary consequence of the accessibility and availability of the substrate to the enzyme at the interface. How a phospholipase $A_2$ molecule at the interface optimizes the catalytic turnover can be operationally viewed as follows.[26] Since the catalytic site of secretory $PLA_2$ is about 1.5 nm away from the surface of the protein, it is likely that the bound enzyme must dislodge the substrate monomer from the interface to the active site without bringing the substrate in contact with the aqueous phase. It appears that this is achieved by desolvation of the microinterface between the enzyme and the interface where the substrate is localized.[25]

*The 'controversies':* There is a general consensus on the existence of the E to E* step as a prelude to interfacial catalytic turnover (Fig. 1). Thus, depending on the equilibrium and kinetic properties, this step may or may not influence the observed steady state reaction rate. Many practical and theoretical difficulties arise because, for a quantitative interpretation of interfacial catalysis, one must keep track of the fraction of the enzyme in the interface, its residence time at the interface, and the local concentrations of the interacting species (substrate, products, inhibitor, calcium) that the bound enzyme 'sees' at the interface. Based on such considerations, for the kinetic analysis it is often necessary to have a detailed quantitative knowledge of the distribution and dynamics of the exchange of the enzyme, substrate, products, and other additives such as surface diluents, activators and inhibitors. Under most of the commonly used conditions for the assay and kinetic analysis, such variables are not controlled. Also in most published analyses of interfacial catalysis[23,27] such variables and the constraints of the dynamics of the substrate and products are not explicitly considered. In short, most, if not all, of the observed anomalies in the interfacial kinetics of $PLA_2$ can be resolved within the general paradigm of the scheme in Fig. 1 if proper attention is paid to the factors regulating the E to E* equilibrium.[14,26] Indeed, for such kinetic analyses it is not necessary to make *ad hoc* assumptions about slow 'penetration' of the enzyme into the interface, nor about dimerization or acylation of $PLA_2$ at the interface. Also it has been experimentally demonstrated that such events do not occur and are not required for the full catalytic activity of $PLA_2$ at the interface.[14,15,18,28–30]

### The steady state condition at the interface

The scheme in Fig. 1 is deceptively simple. It is a remarkably versatile kinetic representation of interfacial catalysis.[14,15] For example, reaction progress curves with virtually any shape can be generated within the constraints of this scheme. This is because a rigorous description of the overall progress of the reaction requires a consideration of not only what the enzyme 'sees', but also how its local environment changes with time. Such considerations include not only the explicit terms in the scheme, but also the implicit constraints that control the 'local' concentration of the substrate and products that the enzyme 'sees' as the reaction progresses. In short, the organization and dynamics of all the molecular species at the interface control the overall effective rate of interfacial catalysis, that is, not only the factors governing the binding of $PLA_2$ to the substrate interface but also the lateral distribution and the rate of exchange of the substrate, products and the enzyme between the interfaces of the aggregates present in the reaction mixture. Some of these considerations are illustrated by the example discussed next.

*Constraints on interfacial catalysis on micelles:* Mixed micelles of phospholipids and detergents have been used extensively as substrates for $PLA_2$.[5,27] They are attractive in the sense that a linear initial rate of hydrolysis is observed for several minutes. On this basis it has been assumed that the substrate concentration that the enzyme 'sees' corresponds to the total substrate in the reaction tube which remains constant for several minutes. This may be so, however as illustrated in Fig. 2 and elaborated below there is no basis for such an assumption. The major difficulties in the interpretation of the kinetics of hydrolysis of mixed-micelles by $PLA_2$ arise from the fact that the rate of hydrolysis depends not only on the nature of the enzyme and the substrate, but also on the nature and the mole fraction of the detergent which acts as a 'diluent' for the substrate and influences the surface charge density, dispersity, and dynamics of the components. The following kinetic and equilibrium consequences of such factors deserve consideration:

(a) In order to compare catalytic activities of phospholipases $A_2$ from different sources, one must optimize the properties of the interface for each enzyme. In effect, it is very unlikely that a single set of experimental conditions using mixed micelles can be used to compare the rates of different enzymes.

(b) It is often assumed that by increasing the bulk mole fraction of the detergent, the substrate is surface diluted.[27] Even after correction for the intermicellar concentration, this assumption would be valid only under equilibrium conditions and only if the enzyme can bind equally well to micelles of the detergent as well as to the mixed micelles.

(c) For the validity of the surface dilution under the kinetic conditions, it is also necessary to assume that the rate of replenishment of the substrate on the enzyme-containing micelles is rapid on the time scale of the hydrolysis of only a small proportion of the substrate molecules in the enzyme containing mixed-micelle.[15,19] Otherwise, the observed steady state rate will depend on the kinetics of phospholipid exchange between mixed micelles.

Since such microscopic constraints for the steady-state condition are not satisfied, it is not possible to interpret the kinetics on mixed-micelles according to the Michaelis–Menten formalism. For example as shown in Fig. 2, consider what the enzyme 'sees' during the course of hydrolysis of the micelle to which it is bound. The steady-state assumption would be valid on the microscopic scale

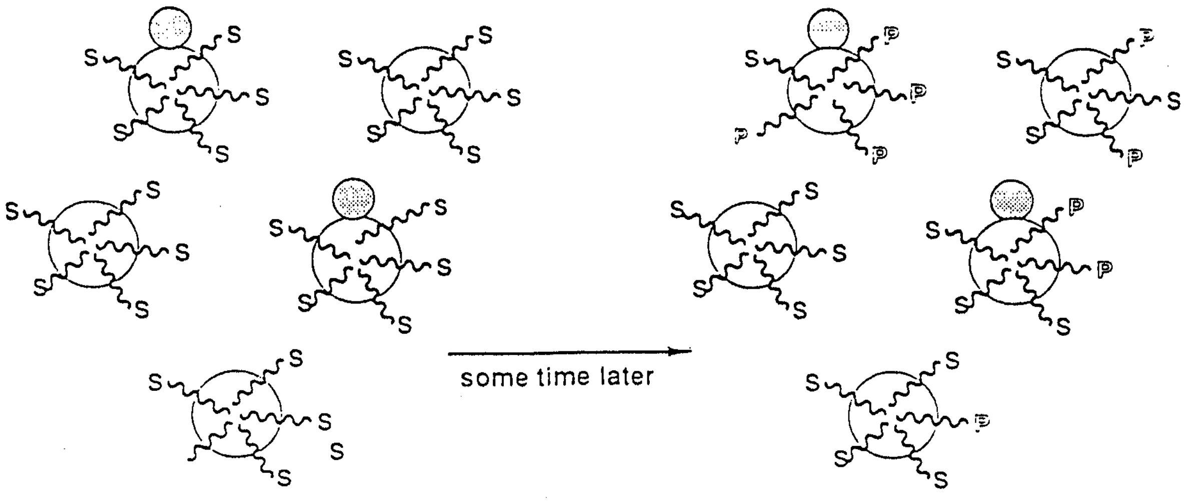

FIG. 2. Schematic illustration of the microscopic events during the course of hydrolysis of micelles under the conditions where the enzyme remains bound to the interface and the substrate exchanges slowly on the time scale of hydrolysis of the substrate molecules present in the micelle. Under such conditions of 'slow replenishment', different enzymes will be in different environments and the effective rate of hydrolysis will be limited by the rate of replenishment.

of a micelle, if and only if the rate of replenishment of the substrate and the rate of removal of products is rapid enough for only an insignificant fraction of the substrate molecules present in the enzyme-containing micelle to be hydrolysed. In a micelle of aggregation number 50 that contains a bound phospholipase $A_2$ with $k_{cat}$ of 300 $s^{-1}$,[15] hydrolysis of ten substrate molecules would change the surface concentration of substrate by 20% in 30 ms. Thus, a true steady state rate in such enzyme-containing micelles can be achieved only if the rate of replenishment of the substrate and the rate of removal of the product from the micelles is rapid on the time scale of 30 ms.

There are two possible mechanisms for the replenishment of the substrate that the bound enzyme 'sees'. If the rate of desorption of the enzyme from the interface is rapid on the 30 ms time-scale, then it would leave the micelle before a significant fraction of the substrate has been hydrolysed. Direct measurements demonstrate that this is not the case since the rate constant for the desorption of the enzyme from the interface is smaller than 0.0002 $s^{-1}$.[19,28] The other possibility is that the replenishment of the substrate occurs by the intermicellar exchange of phospholipids. For long chain phospholipids, the intermicellar exchange involving passage through the aqueous phase occurs only on the time scale of hours. The rate of transfer of phospholipids by a collisional process involving the fusion—fission events, occurs on the time scale of 0.1 to 30 s depending upon the concentration of the micelles,[31] and this is probably the reason why the rate of hydrolysis on micelles increases with the concentration of micelles. It is also pertinent to note that evidence about the fast intermicellar exchange of phospholipids in mixed micelles should not be based on the measurements of the exchange rate of the detergent, but rather on the exchange rate of phospholipids from the enzyme-containing micelles.

Based on such considerations, most observations on the interfacial kinetics by $PLA_2$ on mixed micelles cannot be used to develop a detailed description of the fundamental catalytic properties. The crux of the problem is that the substrate is present in micelles that have a relatively small aggregation number and low exchange rate compared to the catalytic turnover rate. The kinetic analysis of any system requires a perfect mixing of all reactants so that every enzyme, on the average, 'sees' the same environment of substrate and product as in the bulk concentration at any particular time along the progress curve. In solution enzymology, rapid mixing is normally not a concern because the solitary monomeric reacting species diffuse in a common and uniform aqueous environment. However, during interfacial catalysis this is not necessarily the case, and therefore special care has to be taken to ensure that all enzymes operate in a common and uniform environment as the reaction progresses.

*Interfacial catalysis within explicitly defined constraints of organization and dynamics on anionic vesicles:* Our approach for obtaining a rigorous quantitative description of the kinetics of interfacial catalysis is based on the premise that by studying the action of enzyme on relatively large substrate aggregates, such as vesicles, and by eliminating the inter-aggregate exchange of the interacting species, it is possible to rigorously describe the microenvironment of the bound enzyme. As developed in this section, such constraints on the dynamics of all the interacting species can be accomplished on vesicles of anionic phospholipids, where, under suitable conditions, the exchange of the enzyme, substrate, and products is negligible on the time scale of the entire progress curve.[15–20,32–35] Thus as elaborated below, the environment that the bound enzyme 'sees' can be rigorously described.

The molecular organization and dynamics of phospholipids in bilayer vesicles is reasonably well constrained (Fig. 3), which permits an unequivocal description of interfacial catalysis under well characterized experimental boundary conditions.[15] Thus, imagine using vesicles, containing typicallly 10 000 to 200 000 phospholipid molecules, to which a phospholipase $A_2$ molecule binds with such high affinity that once bound it does not leave the vesicle. Two scenarios unfold under the conditions where there is at most one enzyme on each enzyme-containing vesicle. On a large vesicle, 10% of the substrate will be hydrolysed in about one minute by a single bound enzyme molecule. Therefore, it is possible to satisfy the steady state reaction condition on the microscopic level for this duration when the mole fraction of substrate that the bound enzyme 'sees' would remain close to one. In contrast, on a small vesicle containing about 5 000 phospholipids in the outer monolayer, due to a relatively rapid decrease in the mole fraction of substrate and accumulation of the reaction products, only a first order reaction progress curve is observed as the steady state concentration of E*S and the resulting steady state rate will decrease at each successive time point in the reaction progress curve. Vesicle of different sizes and narrow size dispersity can be prepared by the extrusion method. Under such conditions each enzyme-containing vesicle behaves identically in time and all enzymes sample a common environment at all points during the reaction progress.[15] The system has a kind of phase coherence, and therefore the total product formed as a function of time is obtained by simply adding together the contribution of products from

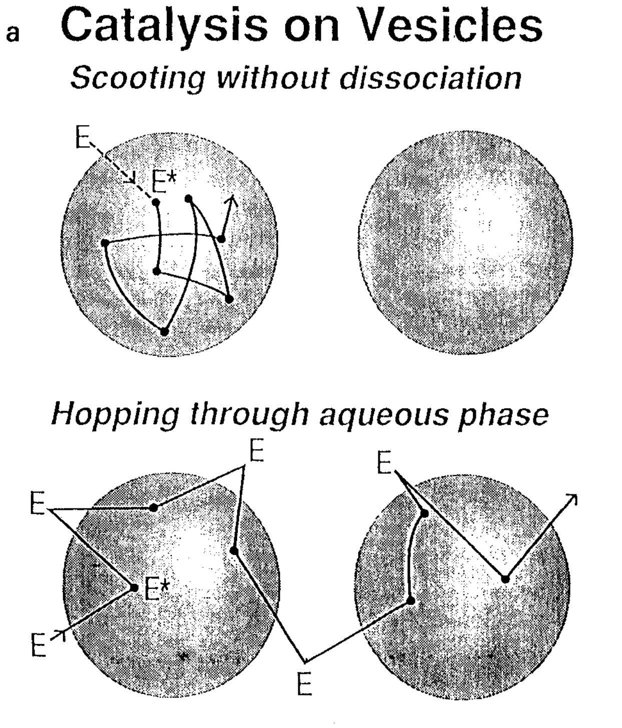

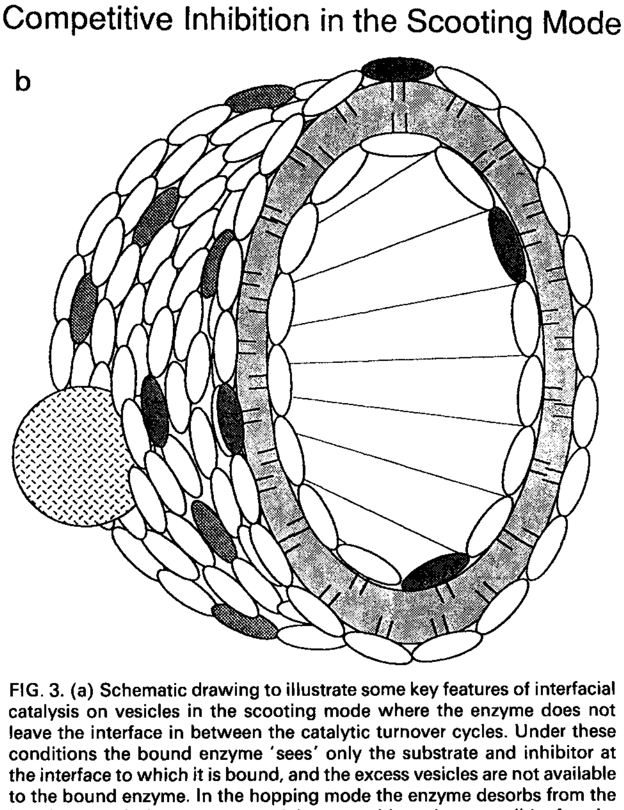

FIG. 3. (a) Schematic drawing to illustrate some key features of interfacial catalysis on vesicles in the scooting mode where the enzyme does not leave the interface in between the catalytic turnover cycles. Under these conditions the bound enzyme 'sees' only the substrate and inhibitor at the interface to which it is bound, and the excess vesicles are not available to the bound enzyme. In the hopping mode the enzyme desorbs from the interface, and thus excess vesicles are ultimately accessible for the hydrolysis. (b) Another schematic representation of $PLA_2$ scooting on the vesicle surface containing substrate and inhibitors.

each of the enzyme-containing vesicles. This is true even though each enzyme molecule operates in its own 'isolated world' of a single vesicle. This highly processive reaction in which the enzyme remains bound to the vesicle during thousands of catalytic turnover cycles is termed scooting.

If the enzyme does not bind tightly to the vesicle it may hop from one vesicle to another during the reaction. Such hopping must be eliminated since the kinetic results under these conditions cannot be readily interpreted. For example, after the initiation of the reaction, an enzyme may leave a partially hydrolysed vesicle and bind to a vesicle that has not 'seen' enzyme previously. Alternatively, another enzyme may leave a vesicle shortly after binding to it and bind to a vesicle that has been completely hydrolysed. Clearly, hopping among vesicles gives a scrambling of the time of reaction and invalidates the kinetic analysis. Even if the hopping occurs rapidly, under such conditions one must take into consideration the contribution of the 'on' and 'off' steps for the enzyme to and from the interface in the catalytic turnover cycle,[14,15] which may not necessarily be once per catalytic cycle.

Other attractive features of interfacial catalysis in the scooting mode may also be noted.

(a) The E to E* equilibrium is completely in favour of E*,[14] and the hydrolysis starts immediately after the addition of $PLA_2$.[32]

(b) The binding isotherms of $PLA_2$ to vesicles are quantitatively interpreted in terms of the E + nL to E* equilibrium,[26,33,36] where binding of one enzyme molecule to the interface requires n lipid molecules.

(c) Binding of $PLA_2$ to the bilayer interface does not require occupancy of the active site of the enzyme by a substrate, inhibitor or calcium, which establishes that the binding of the enzyme to the interface and the catalytic cycle are separate steps.[16,18,26]

(d) Over 30 different $PLA_2$ from a variety of sources also exhibit catalysis in the scooting mode not only on DMPM vesicles[18] but also on covesicles of zwitterionic and anionic phospholipids.[17] This suggests that a highly processive catalytic turnover at the interface is a general property of anionic interfaces.

(e) The gross organization of the bilayer is not altered on binding of $PLA_2$ to the outer monolayer of anionic phospholipid vesicles.[14,15]

(f) In bilayers, the reaction sequence in the aqueous phase is neglected because the concentration of solitary substrate molecules in the aqueous phase is exceedingly small (< 100 pM) compared to the apparent affinity of the enzyme for the monomeric substrate.

(g) The half-time for transbilayer movement (flip-flop) of phospholipids is of the order of several hours.[21]

(h) The intervesicle exchange of the substrate and the products is negligible on the time scale usually employed for the kinetic studies.[24,31]

(i) The rate of lateral diffusion of phospholipids in vesicles is at least ten times faster than the rate of catalytic turnover.[14]

(j) The integrity of the vesicles is maintained because under suitably chosen conditions the rate of fusion of vesicles is negligibly small.[32]

In addition, the products of hydrolysis of long chain phospholipids also form bilayers.[37] Thus, the integrity of the substrate bilayer is maintained even when all the substrate molecules in the outer monolayer of the target vesicles are hydrolysed, and contents of the inner aqueous compartment of vesicles are not released even when the bilayer surface is substantially covered by $PLA_2$.[15]

These observations demonstrate that not only is the bilayer organization retained during and after the hydrolysis of vesicles by $PLA_2$, but within the constraints of the organization and dynamics of phospholipids in bilayer vesicles it is possible to rigorously describe the kinetics of interfacial catalysis in the scooting mode on anionic vesicles, the steady state initial rate of hydrolysis is observed for several minutes under these conditions.[32] Similarly, by a yet unknown mechanism, the contribution of the parallel kinetic processes without compromising the underlying catalytic mechanism for the enzyme.

*Lipid transfer between vesicles:* Owing to the finite size of vesicles, in the absence of intervesicle exchange of phospholipids, the initial steady state rate in the largest vesicles can be maintained only for about a minute. It can however be extended by promoting intervesicle transfer of phospholipids as accomplished in two different ways for DMPM vesicles. At calcium concentrations greater than 1.5 mM, the half-time for fusion of DMPM vesicles is of the order of a few seconds. Thus, even with small vesicles, the steady state initial rate of hydrolysis is observed for several minutes under these conditions.[32] Similarly, by a yet unknown mechanism, polymyxin B increases the rate of intervesicle transfer of DMPM molecules, such that in the presence of 1 $\mu$M polymyxin B, the steady state initial rate of hydrolysis is observed for several minutes.[19] The initial enzymatic rate observed under these conditions corresponds to the steady-state rate at mole fraction 1 of the substrate at the interface. As described later such conditions are very useful for kinetic characterization of $PLA_2$ activity and inhibition because all the enzyme remains at the interface.

*Hydrolysis of zwitterionic vesicles:* Compared to anionic vesicles, the affinity of most $PLA_2$ for zwitterionic vesicles is poor, and it increases in the presence of anionic additives. For example the dissociation constant, $K_d$, for pig pancreatic $PLA_2$ from DTPC vesicles is $>10$ mM, more than $10^{10}$-fold larger than the dissociation constant from DTPM or DMPM vesicles ($K_d < 1$ pM). This difference is apparent in reaction progress curves for the hydrolysis of DMPC vesicles[14] where a lag period is observed. The duration of the lag depends on the presence of lipophilic additives and on the gel–fluid transition properties of the bilayer. The lag is shortest and the percentage hydrolysis at a single time point is highest (apparent activation) at the gel–fluid phase transition temperature of phosphatidylcholine bilayers. Such a lag is not observed in phosphatidylcholine vesicles that contain the products of hydrolysis or other anionic amphiphiles. This is due to an increase in the fraction of the bound enzyme.[36,38] Also the $K_d$ for E* on DTPC vesicles remains the same ($>10$ mM) either at, below, or above the phase transition temperature.[36,39] Thus the shape of the entire progress curve, including the lag period and the apparent activation, can be quantitatively accounted for in terms of the product-dependent shift in the E to E* equilibrium.[14] As fatty acid molecules are formed, the vesicles take on an anionic character and this causes an increase in the fraction of vesicle-bound enzyme with a concomitant increase in the reaction velocity. Even in the presence of the products of hydrolysis the $K_d$ on the ternary vesicles is of the order of 0.1 mM. Therefore the bound enzyme hops between vesicles. As discussed earlier, under these conditions the 'on' and the 'off' steps contribute to the catalytic turnover period. Such anomalous kinetics observed with zwitterionic vesicles makes them rather unsuitable for assay of $PLA_2$ activity and inhibition.

*Uses of interfacial catalysis in the scooting mode:* The unique features of catalysis in the scooting mode on anionic vesicles permit quantitative characterization of virtually all aspects of interfacial catalysis. Such studies with DMPM vesicles have unequivocally demonstrated that secretory $PLA_2$ from virtually all sources are fully catalytically active as monomers.[18] In addition, the entire reaction progress curve has been characterized to obtain values of the interfacial rate and equilibrium parameters for the pancreatic $PLA_2$.[15] This provides a basis for evaluating substrate specificity,[17] specific competitive inhibitors,[16,20,35,40] effect of activators such as polymyxin B[19] and calcium,[16] and the kinetics of covalently modified enzymes.[16,18,37]

Through such studies, it has also been demonstrated that the effects of many previously reported phospholipase $A_2$ inhibitors are due to a shift in the E to E* equilibrium toward the enzyme in the aqueous phase.[16,41] Similarly, the anomalous kinetics at the gel–fluid transition or at isothermal phase transition in zwitterionic bilayer is due to a shift in the E to E* equilibrium.[14,33,36–39] These observations demonstrate that even though the organization and dynamics of the amphiphiles in the interface has a profound influence on the E to E* equilibrium, the primary catalytic parameters for the turnover in the interface are virtually insensitive to the nonspecific organizational perturbations. Through such protocols that unequivocally distinguish the kinetic contribution of the E to E* step from the steps involved in the catalytic turnover cycle, it should be possible to carry out a variety of structure–activity correlations not only with substrates and inhibitors, but also with site-directed mutants designed in an effort to understand the role of specific amino acid residues in the catalytic and interfacial events.

### Assay of phospholipase $A_2$ activity and inhibition in the scooting mode

The major problems encountered in the assay of phospholipase $A_2$ activity are due to the inability to control the E to E* equilibrium. To some extent this equilibrium depends on the organization and dynamics of the interface, therefore activities of enzymes from different sources cannot be compared readily on certain interfaces. Also the presence of lipophilic impurities can shift the E to E* equilibrium and thus modulate the apparent activity. This difficulty can be minimized to a certain extent by using sufficiently high bulk substrate concentrations so that all the enzyme molecules are bound to the interface. However, at interfaces containing zwitterionic phospholipids the apparent $K_d$ for $PLA_2$ is often in the millimolar range. Thus, the problem becomes particularly acute with radiolabelled or fluorescently labelled substrates which are used typically in the submicromolar concentration range. Under such conditions only a fraction of the total enzyme is bound, and this fraction could change significantly in the presence of impurities.[16,40,41] Many phospholipase $A_2$ assays suffer from this problem, although with proper considerations the micelle-based assays have been used to identify relatively potent specific inhibitors of $PLA_2$.[42] In addition, a major problem with such assays for $PLA_2$ inhibitors is that not only do they give false-positives[16] but as elaborated earlier in this article, with such assays it is virtually impossible to obtain the primary kinetic and inhibition parameters which have well established mechanistic significance.[15]

The difficulties mentioned above can be resolved almost completely by using substrates with anionic head groups. Radiometric, fluorescence or absorbance based assays using appropriately labelled anionic and zwitterionic phospholipids have been reported.[43–46] Such assays permit the continuous measurement of $PLA_2$ activity in as little as 5 $\mu$l of crude blood plasma, corresponding to a few picograms of pancreatic or snake venom enzyme. However, adoption of such assays for inhibition studies requires proper consideration of the background quenching by the test solutes, and the various binding, partitioning and solubility equilibria. Similarly, the assay system consisting of radiolabelled "autoclaved *Escherichia coli*" has never been fully characterized. These dispersions contain a relatively high mole fraction of anionic phospholipids such as cardiolipin and phosphatidylethanolamines. However, only a relatively small amount of the substrate (submicromolar range) is used for the assay of the $PLA_2$ inhibition, which creates difficulties as it gives false-positives due to a solute-dependent shift in the E to E* equilibrium.

Virtually all the problems associated with the assay of $PLA_2$ activity and specific inhibition are obviated by the use of DMPM vesicles under constant pH conditions,[15–20,40,47] where $PLA_2$ activity from virtually all sources and their mutants can be assayed under identical conditions on the same interface in the absence of additives. Since the $K_d$ for E* on DMPM vesicles is in the picomolar range, the bulk substrate concentrations in the micromolar range are adequate to ensure that all the enzyme remains at the interface, and yet there is sufficient substrate available to the bound enzyme to allow hydrolysis to proceed for several minutes under steady state conditions. Such small bulk concentrations of substrate also ensure that the inevitable background from the probes remains low. On such anionic interfaces, lipophilic impurities have little effect on the E to E* equilibrium, therefore the activity is not perturbed by nonspecific additives incorporated up to 0.2 mole fraction in the interface. This is a particularly important consideration because the mole fraction of an additive in the interface depends not only on its absolute concentration but also on its bilayer/-water partition coefficient. For example, with a substrate concentration of 1 $\mu$M, only a 0.4 $\mu$M concentration of a hydrophobic additive would be sufficient to bring the interface mole fraction to 0.3. Such problems are minimized in the pH-stat titration assay where the bulk concentration of substrate can be 1 mM without significant background rates. As discussed in the next section, this assay can be used for quantitative and mechanistic characterization of specific competitive inhibitors without any false-positives.

Other distinct advantages of protocols for catalysis in the scooting mode include:

(a) The same assay can be used for phospholipase $A_2$ from virtually all sources.[18]

(b) It can be used to establish the presence of a low abundance $PLA_2$ impurity.[28]

(c) Based on the same general principle, the effects of lipophilic impurities on the E to E*

equilibrium, if any, can be directly monitored by determining the extent of hydrolysis per enzyme.[15,32,47]

(d) Effects associated with the partitioning of inhibitors can also be discerned by catalysis in the scooting mode.[40] Since the affinity of secretory $PLA_2$ for anionic vesicles is very large, dilution of the reaction mixture would influence the rate of hydrolysis only if the inhibitor was poorly partitioned in the interface.

### Characterization of specific inhibitors

*Criteria for competitive inhibition:* Within the constraints of the organization and dynamics of the bilayer, interfacial catalysis in the scooting mode can be adopted to obtain quantitative information about the whole Michaelis–Menten space, including the characterization of competitive inhibitors.[14,15] The primary considerations and the basic protocols are outlined below.

*Rates of hydrolysis:* The ratio of the initial rates of hydrolysis in the scooting mode in the absence ($v_o$) and in the presence ($v_I$) of a competitive inhibitor at mole fraction $X_I^*$ at the interface is given by:

$$\frac{v_o}{v_I} = 1 + \left( \frac{1 + \dfrac{1}{K_I^*}}{1 + \dfrac{1}{K_M^*}} \right) \left( \frac{X_I^*}{1 - X_I^*} \right) \quad (1)$$

Similarly, the ratio of the turnover numbers under the first-order kinetic conditions ($N_s k_i$) in the presence and in the absence of a competitive inhibitor at mole fraction $X_I^*$ is given by:

$$\frac{(N_s k_i)_o}{(N_s k_i)_I} = 1 + \left( \frac{1 + \dfrac{1}{K_I^*}}{1 + \dfrac{1}{K_P^*}} \right) \left( \frac{X_I^*}{1 - X_I^*} \right) \quad (2)$$

Here $N_s$ is the number of substrate molecules on the outer monolayer of the vesicles (related to the vesicle size), and $k_i$ is the first-order relaxation constant for the hydrolysis of the $N_s$ molecules.[14] The terms $K_M^*$, $K_I^*$ and $K_P^*$ expressed as mole fractions have their usual meaning in the context of the Michaelis–Menten formalism adopted for catalytic turnover at the interface. The validity of these relations has been elaborated[15] and experimentally demonstrated[16–20] elsewhere. However, intuitively it makes sense that under initial velocity conditions in large vesicles where the mole fraction of substrate is close to one, the inhibitor competes with the substrate and thus the ratio of velocities depends on the relative magnitudes of the $K_M^*$ for the substrate and the $K_I^*$ values for inhibitors (equation **1**). With small vesicles under the first-order kinetic conditions, the mole fraction of the products increases rapidly, and the relative rate equation contains the $K_P^*$ for products as well as the $K_I^*$ since the inhibitor competes with the product for the binding to the enzyme (equation **2**).

Based on these relationships a plot of $v_o/v_I$ versus $X_I^*/(1 - X_I^*)$ is linear (cf. Dixon plot), with a y-intercept of one, and the value of $X_I(50)$, the mole fraction of the inhibitor for 50% inhibition is obtained from the slope. For a series of inhibitors, the $X_I(50)$ values are related to $K_I^*$. The value of $K_I^*$ or $K_M^*$ can be calculated if one or the other term is obtained independently.[16] Similarly, from the effect of an inhibitor on the first order curve it is possible to obtain $n_I(50)$, the mole fraction for 50% decrease in the value of $N_s k_i$. For the action of pig pancreatic phospholipase $A_2$ on DMPM vesicles, $K_M^* = 0.35$ mole fraction and $K_P^* = 0.025$ mole fraction.[16] The value of $K_M^*$ is in the same range as the concentration of the substrate in the interface. Therefore, a potent inhibitor like MJ33, $K_I^* = 0.0008$ mole fraction[20] would have a significant inhibitory effect even if one inhibitor molecule is present per 1 000 substrate molecules in the interface.

*Kinetic proof for competitive inhibition:* The ratio

$$\frac{(N_s k_i)_o}{v_o} = \frac{\left(1 + \dfrac{1}{K_M^*}\right)}{\left(1 + \dfrac{1}{K_P^*}\right)} = \frac{\left(\dfrac{1}{X_I(50)} - 1\right)}{\left(\dfrac{1}{n_I(50)} - 1\right)} \quad (3)$$

is particularly diagnostic because it provides a kinetic proof for the competitive nature of the inhibition of interfacial catalysis in the scooting mode. The $(N_s k_i)_o/v_o$ ratio can also be obtained by independent kinetic methods; either from the analysis of the whole progress curve,[16] or from the $K_M^*$ and $K_P^*$ values determined independently.[20]

*Concentration dependence:* The concentration dependence of an inhibitor is always viewed in the context of the substrate interface. For a very hydrophobic inhibitor which partitions essentially completely in the interface, the $X_I^*$ value can be calculated on the basis of the bulk inhibitor and substrate concentration as $[I]/([I] + [S])$. For inhibitors which are poorly partitioned it is necessary to consider the fraction of the inhibitor that is in the interface. Although partition coefficients can be determined independently, the effect of such factors can be qualitatively judged on the basis of experiments in which the dilution of the reaction mixture has a significant increase in the effective $X_I(50)$ values if the inhibitor is poorly partitioned in the bilayer (see also later). Since the E to E* equilibrium is not noticeably influenced under these conditions, such dilution experiments can be quantitatively interpreted to obtain the values of the partition coefficient and true $X_I(50)$ values.

*Tight binding phospholipid analogues:* So far two classes of potent inhibitors of $PLA_2$ have been identified (Fig. 4). In molecules like MG14 or MJ33 there is a phosphonate or a phosphate group which presumably resembles the tetrahedral transition state formed during the hydrolysis of the ester bond in the *sn*-2-position.[20,40] The X-ray structure of MG14 bound to $PLA_2$ reveals a coordination of one of the phosphonate oxyanions to the calcium, and the other oxygen to a protonated imidazole ring of the active site histidine. In addition one of the *sn*-3-phosphate oxygens of MG14 is also bound to the calcium. Replacement of the *sn*-2 ester linkage by an amide also gives quite potent inhibitors,[20,42] and the oxygens on the *sn*-2- P=O or C=O groups are coordinated directly to calcium at the active site.[7] Similarly, the $P—O^-$ of the *sn*-2-phosphate interacts with the cationic charge on the protonated histidine.[6] Binding of these inhibitors requires the presence of calcium, although barium does not support such binding.[20]

These interactions at the active site are consistent with the X-ray crystallographic results on the co-crystals[6,7,9] which also demonstrate that the *sn*-3-phosphate is liganded to the calcium ion and is also hydrogen-bonded to Tyr-69. However, such an interaction may not contribute to the stability of the complex because the $K_I^*$ values for inhibitors like MJ33, which do not have a *sn*-3-phosphate group, are in fact somewhat smaller than those for inhibitors containing the *sn*-3-phosphate.[20] In addition, the N—H of the amide forms a hydrogen-bond with the imidazole N—H of His-48. Thus, opposite effects of pH are predicted and observed[44]: $K_I^*$ for amides decreases at higher pH, whereas the $K_I^*$ for the *sn*-2-phosphates or phosphonates increases at lower pH.

*Lipophilic solutes:* Lipophilic solutes have a variety of effects on the integrity of vesicles. Such effects can be unambiguously discerned by monitoring the scooting kinetics under the first order conditions. Here, any significant effect on the size of vesicles obtained from the first order progress curve would be a caution flag against its efficacy as a $PLA_2$ inhibitor. As summarized in Table 1 many of the inhibitors reported in the literature have been shown[16] to be nonspecific because they reduce the rate of hydrolysis by promoting the desorption of the bound enzyme.

With the criteria summarized in this section a broad range of specific competitive inhibitors of $PLA_2$ have been kinetically characterized[20,32,40] and these can be unequivocally distinguished from other nonspecific inhibitors.[16,38,48] Many of the competitive inhibitors identified so far are effective at $<0.001$ mole fraction in the interface, they are water soluble (as monomers or they disperse as

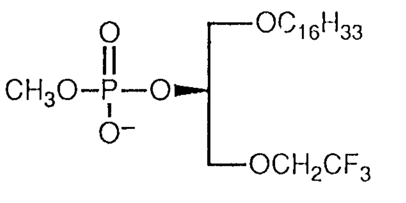

MJ33

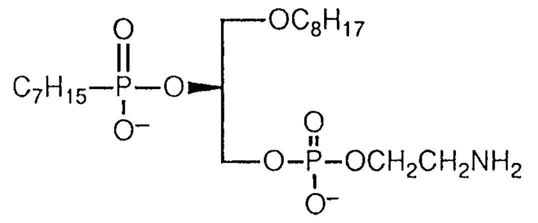

MG-14

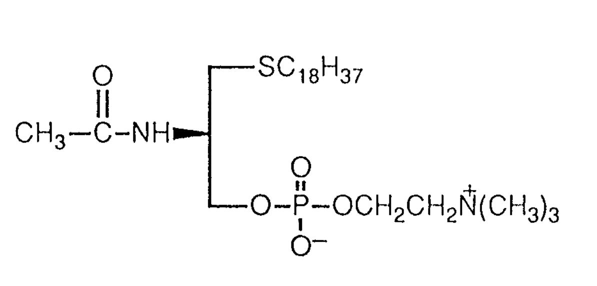

RM-2

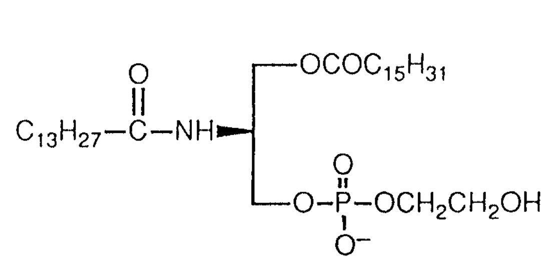

H-18

FIG. 4. Structures of four potent competitive inhibitors of $PLA_2$: MJ33, RM-2, H-18, and MG-14 with $K_I$ values 0.0008, 0.0028, 0.0016 and 0.001 mole fractions, respectively, for pig pancreatic phospholipase $A_2$. See reference 18 for details.

micelles), and they partition readily and essentially completely into the bilayer. Since these inhibitors probably do not cross the cell membrane, they have had little use as pharmacological agents. However, recently we have successfully delivered MJ33 in intact lung by taking advantage of the fact that the liposomes containing inhibitors are taken up by intact cells. MJ33 delivered in this fashion had no effect on the arachidonate cascade, although it selectively blocked the secretory $PLA_2$ activated during ischaemia.[49]

*Independent methods to measure $K_I^*$:* Besides the kinetic methods described above, two other independent methods have been used to quantitatively characterize the binding of inhibitors to the active sites of $PLA_2$.

*Use of ligands:* The rate of chemical modification of histidine-48 in the catalytic site of secretory $PLA_2$ by an alkylating agent is modulated in the presence of ligands that bind to the catalytic site.[16] Such ligands include calcium, specific inhibitors, $PLA_2$ reaction products, and substrate analogues. The kinetics of alkylation of phospholipase $A_2$ bound to a neutral diluent, such as 2-hexadecylglycerophosphocholine, is not modulated because such amphiphiles have poor affinity for the catalytic site. Thus, from the relative rates of alkylation of the enzyme bound to a neutral diluent in the absence and in the presence of a suitable ligand, it is possible to obtain the values of $K_{Ca}$, $K_I^*$, $K_P^*$, and $K_S^*$, respectively.

*Spectroscopic methods:* Under suitable conditions, binding of a ligand to phospholipase $A_2$ present at the interface of a neutral diluent produces perturbations in the UV and fluorescence emission spectral properties of the enzyme. For example, when pig or bovine pancreatic $PLA_2$ bound to the interface of a neutral diluent is titrated with an increasing mole fraction of an inhibitor, characteristic UV spectral changes are observed in the 280–300 nm region. Such titration curves can be used to obtain $K_I^*$ values, which are identical to those obtained by the protection from alkylation method.

*Inhibition of the hydrolysis of water-soluble monomeric substrates:* Short chain zwitterionic phospholipids below their critical micelle concentrations are also hydrolysed by $PLA_2$, and the hydrolysis is inhibited by the specific competitive inhibitors.[50] According to equation **4**:[22]

$$I(50) = K_I^{kin}\left(1 + \frac{[S]}{K_M}\right) \qquad \textbf{(4)}$$

it is possible to obtain values of $K_I^{kin}$ (the KI for the inhibition determined in kinetic studies) and $K_M$ from the dependence of I(50) on [S]. The $K_I^{kin}$ values obtained under these kinetic conditions are always significantly smaller than the dissociation constant for the enzyme inhibitor complex in the aqueous phase. However the $K_I^{kin}$ values are similar to the values of the effective dissociation constants obtained for the enzyme at the interface of a neutral diluent.[51] These results show that even if the substrate molecules are dispersed as solitary monomers, during the course of their hydrolysis the pig pancreatic phospholipase $A_2$ is in a state that resembles the enzyme bound to the interface. One of the ways in which this could happen is that binding of a substrate molecule to the active site promotes (nucleates) aggregation of additional substrate molecules so as to achieve appropriate hydrophil—liphophil balance. Thus the ES complex is stabilized as a larger aggregate with additional amphiphiles present in the medium.

*Binding to the interface versus binding to the active site:* Micelles of alkylphosphocholines have been used as amphiphile models in physical studies of bound $PLA_2$. Protection from alkylation studies show that these amphiphiles have a modest affinity for the active site, $K_I^* = 0.65$ mole fraction.[18] Two processes must be considered. The bulk concentration of the amphiphile modulates the fraction of the enzyme bound to micelles (E*). Since the mole fraction of the amphiphile at the interface is always one, about 60% of the enzyme would be in the E*I form and 40% in the E* form in the presence of an excess of bulk amphiphile. Thus virtually all such results on the physical studies at saturating bulk concentrations of the amphiphile contain information about the E* as well as the E*I form of the enzyme.

### Screening strategy for phospholipase $A_2$

As discussed in this review, analysis of inhibitors of $PLA_2$ in the scooting mode eliminates the kinetic complications due to the perturbation of the E to E* step. This analysis is not designed to detect inhibitors that bind to the interfacial recognition site of $PLA_2$ in the aqueous phase and prevent its association with the substrate interface. This is because the affinity of the enzyme toward anionic interfaces is very large and therefore the concentration of the enzyme in the aqueous phase is insignificant. Under pharmacological conditions it is conceivable that this may not be the case. General consequences of the possible equilibria at the interface under such conditions are elaborated below.

In principle, a phospholipase $A_2$ inhibitor could interact either in the membrane phase with E* or in the aqueous phase with E. Thus, in order to develop an optimal strategy to search for specific active site-directed inhibitors of $PLA_2$, it is useful to consider a general scheme in which all species partition between the water and membrane phases. In order to identify inhibitors that are likely to function *in vivo*, it is also necessary to consider the factors that apply in the screen versus in man. The minimal scheme in Fig. 5 takes into consideration all the necessary interactions. The equilibrium constants in this scheme are defined as follows:

$$K_I = \frac{[E]_w[I]_w}{[EI]_w} \qquad K_I^* = \frac{[E^*]_w X_I^*}{[E^*I]_w}$$
$$K_d = \frac{[E]_w[A^*]_w}{[E^*]_w} \qquad K_d^I = \frac{[EI]_w[A^*]_w}{[E^*I]_w} \qquad (5)$$
$$K' = \frac{[I]_w}{X_I^*} \qquad \frac{K_d^I}{K_d} = \frac{K'K_I^*}{K_I}$$

It is useful to appreciate the key considerations underlying these definitions:

(a) The concentration terms (in the square brackets) with the w subscript designate the moles of the indicated species per volume of water phase, $V_w$. The asterisks denote species that are bound to the *interface* of the amphiphile phase A. For example, A* designates the moles of amphiphile A that are at the interface and therefore available for enzyme binding, and in general A* will be smaller than the total moles of A in the system, $A_T$. For example, in the screen, only the amphiphile molecules in the outer layer of the vesicles (about 50%) can interact with the enzyme. In man, it is likely that $A^*/A_T$ will be even smaller because only a small fraction of the total A phase is exposed to the enzyme. $X_I^*$ designates the mole fraction of inhibitor in the *total* A phase if the inhibitor can partition into the total A phase. Since the amount of A is typically in large excess over the amount of inhibitor in the A phase, $I_A$, it usually holds that $X_I^* = I_A/A_T$.

(b) $K_I$ and $K_I^*$ denote the dissociation constants for the enzyme-inhibitor interactions in the water phase and A phase, respectively. $K_d$ and $K_d^I$ denote the equilibrium constants for the dissociation of either E* or E*I from the interface (A* phase).

(c) $K'$ is the concentration of the amphiphile at which half of the inhibitor is bound to the amphiphile interface. It is related to the standard partition coefficient for the inhibitor, $P_I = (I_A/V_A)/[I]_w$, by the relationship $f_wK'/[A]_T = (V_w/V_A)/P_I = I/I_A$. Here $V_A$ is the volume of the total A phase, $f_w$ is the fraction of total system volume, $V_T$, that is the aqueous, and $[A]_T = A_T/V_T$.

(d) The last definition is a thermodynamic relationship that follows from the definitions of the equilibrium constants. Also note that the unit of $K_I^*$ is mole fraction, the unit of $K_I$, $K_d$ and $K_d^I$ is moles/l.

Using the above definitions, an expression for the effective dissociation constant for the enzyme—inhibitor interactions is given as follows:

$$K_I^{eff} = \frac{(E + E^*)(I + I^*)}{V_T(EI + E^*I)} = K_I^*[A]_T \frac{\left(1 + \frac{K_d}{[A^*]_w}\right)\left(1 + \frac{f_wK'}{[A]_T}\right)}{\left(1 + \frac{K_d^I}{[A^*]_w}\right)} = \frac{K_I^*[A]_T\left(1 + \frac{K_d}{[A^*]_w}\right)\left(1 + \frac{I}{I_A}\right)}{\left(1 + \frac{EI}{E^*I}\right)} \qquad (6)$$

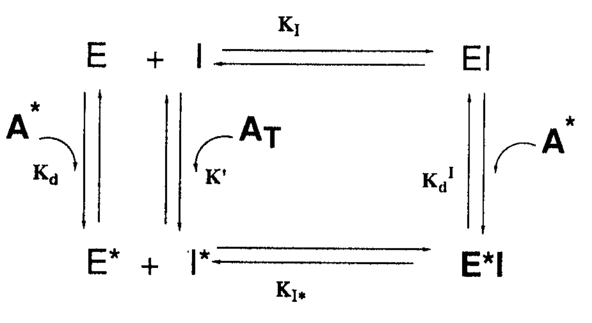

FIG. 5. Interaction scheme to illustrate the partitioning of all enzyme (E) and inhibitor (I) species between the aqueous and membrane phase consisting of the amphiphile (A). The equilibrium dissociation constant of the bound enzyme and the enzyme inhibitor complex from the interface is given by $K_d$ and $K_d^I$, respectively. Similarly, the equilibrium dissociation constants for the inhibitor from the enzyme in the aqueous phase and in the interface are given by $K_I$ and $K_I^*$, respectively. The distribution coefficient for the inhibitor between the aqueous phase and the interface is given by $K'$, i.e., the concentration of the interface at which half of the inhibitor is in the interface. See text for details.

To develop a 'feel' for the interpretation and application of this general equation it is useful to imagine an inhibitor that has a portion that interacts with the active site of the enzyme and another portion that extends away from the enzyme and only interacts with the environment. The first requirement for the design of a potent inhibitor must be to provide maximal interactions in the active site. This part of the inhibitor structure will influence primarily the values of $K_I^*$ and $K_I$. Changes in the remaining portion, however, will have little or no effect on these constants but can influence the efficiency of the inhibitor by changing the partitioning of I and EI between the water and amphiphile (A) phases. It is reasonable to suggest that such changes will affect $K'$ and $K_d^I$ by the same factor, $\beta$. If we compare two inhibitors

**Table 1.** Compounds that had no effect at mole fraction 0.1 on the alkylation and on interfacial catalysis in the scooting mode (Adopted from reference 16)

| |
|---|
| Alkanols ($C_6$ to $C_{14}$ including cis and trans tetradec-9-enol) |
| Fatty acids ($C_8$ to $C_{20}$ including 18:1, 18:2, 18:3 and 20:4), 7,7-dimethyl-eicosadienoic acid, and long chain phenols |
| Local anaesthetics (dibucaine, tetracaine, procaine, benzocaine) |
| Antimalarials (mepacrine and quinacrine) |
| Ketamine, halothane, chloroform, carbon tetrachloride, EMD 21657 |
| Manoalog*, gossypol*, alkylating agents |
| Indomethacin, cortisone and related antiinflammatory agents, alphaxalone, flufenamic acid, phenothiazines |
| Aristolochic acid, plastatin, lipocortin |
| Butyrophenone (U10029A) |
| Alkylammonium salts ($C_{10}$ to $C_{18}$), spermine, putrescine, polymyxin B |
| Antibiotics (gentamycin, tobramycin, streptomycin) |

* A covalent modifier that have similar active—site interactions but differ in their structures outside of the enzyme, which is going to be the most efficient in the screen and in man? If we replace $K'$ and $K_d^I$ in the equation **5** by $\beta K'$ and $\beta K_d^I$ and seek the smallest value of $K_I^{eff}$ as a function of $\beta$, two extreme values are found for either very large or very small $\beta$.

For small $\beta f_w K'/[A]_T$ and $\beta K_d^I/[A^*]_w$:

$$(K_I^{eff})_{\beta_{small}} = K_I^*[A]_T\left(1 + \frac{K_d}{[A^*]_w}\right) = \frac{K_d^I K_I}{K_d K'}[A]_T\left(1 + \frac{K_d}{[A^*]_w}\right) \quad \textbf{(7a)}$$

The expression on the right is obtained by replacing $K_I^*$ with the equivalent collection of constants according to equation **5**.

For large $\beta f_w K'/[A]_T$ and $\beta K_d^I/[A^*]_w$:

$$(K_I^{eff})_{\beta_{large}} = \frac{f_w K_I^*[A^*]_w}{K_d}\left(1 + \frac{K_d}{[A^*]_w}\right) \quad \textbf{(7b)}$$

Small $\beta f_w K'/[A]_T$ implies that $I_A > I$ and small $\beta K_d^I/[A^*]_w$ implies that $E^*I > EI$.

The ratio of the values of $K_I^{eff}$ for small and large $\beta$ is given by equation **8**.

$$\frac{(K_I^{eff})_{\beta_{small}}}{(K_I^{eff})_{\beta_{large}}} = \frac{K_d^I}{(K' f_{A^*})} = \frac{\left(\frac{EI}{E^*I}\right)}{\left(\frac{I}{I_A}\right)} \quad \textbf{(8)}$$

Here, $f_{A^*}$ is the fraction of A that is at the interface, $f_{A^*} = A^*/A_T$. When the ratio in equation **8** is less than one, this implies that the case of small $\beta$ leads to a smaller value of $K_I^{eff}$ and therefore the best inhibitor will be the one with the smallest $K_I^*$ and that partitions mostly into the A phase. Conversely, when this ratio is larger than one, the best inhibitor will be the one with the lowest $K_I^*$ (also the lowest $K_I$ since the change described by $\beta$ does not involve direct enzyme—inhibitor interactions) and that partitions mostly into the aqueous phase. In other words, equation **8** expresses the relative partitioning between the two phases of the enzyme—inhibitor complex and the inhibitor alone, and the phase in which the inhibitor functions most efficiently depends on this ratio.

The ratio in equation **8** is hard to predict and its value will vary from inhibitor to inhibitor. On the one hand, the partitioning of EI into the interface relative to I alone is favoured by the fact that the EI complex can interact with the interface using *both* the regions of I that protrude from the enzyme (effected by $\beta$) and the interfacial binding surface of the enzyme. On the other hand, the partitioning of I into the membrane is favoured over EI by two factors. Firstly, the portion of I that is within the confines of the enzyme will help I, but not EI, to partition into the interface. Secondly, the possible advantage of EI partitioning over I will be reduced by the fact that in man the value of $f_{A^*}$ is expected to be significantly smaller than in the screen.

The ratio given in equation **8** will in general have a different value for every inhibitor and furthermore there is no *a priori* way to predict the value of this ratio. Thus, it is not a general solution to the problem of deciding whether to inhibit the $PLA_2$ in the aqueous phase versus the membrane phase. Based on the following arguments, it is probably best to use a screen in which the phospholipase $A_2$ is operating in the scooting mode. Firstly, in order to search for inhibitors that act in the aqueous phase, it is necessary to use an assay in which the enzyme is not tightly bound to the interface. However, as discussed previously there is precedence demonstrating that such an assay will pick up compounds that function as nonspecific modulators of the E to E* equilibrium.[16] Secondly, it is difficult to predict *a priori* whether an inhibitor that binds to E will function *in vivo*. This is because the degree of inhibition will depend not only on the $K_I^*$ value but also on the affinity of the $PLA_2$ for the various interfaces that exist *in vivo*, and the magnitude of such affinities is not yet known. The scooting mode analysis detects inhibitors that have a low value of $K_I^*$ and such compounds would almost certainly work in man. This is because regardless of where the enzyme is located most of the time *in vivo*, it must go to the interface for the lipolysis reaction.

In the scooting mode analysis, $E^* \gg E$, and it is also very likely that $E^*I \gg EI$ since the binding of the enzyme to anionic interfaces occurs with high affinity. With these inequalities, equation **6** can be simplified to give equation **9**.

$$K_I^{eff} \cong K_I^*[A]_T\left(1 + \frac{f_w K'}{[A]_T}\right) = K_I^*[A]_T\left(1 + \frac{I}{I_A}\right) \quad \textbf{(9)}$$

In man, the term $I/I_A$ is expected to be smaller than one for most compounds based on the following argument. The value of $P_I$ for most organic compounds is larger than 100. In man, $V_A$ and $V_W$ are within a factor of ten of each other, and so it is expected that $I_A > I$ for most inhibitors and equation **9** can be simplified to give equation **10**.

$$(K_I^{eff})^{Man} \cong K_I^*[A]_T^{Man} \quad \textbf{(10)}$$

In contrast, in typical $PLA_2$ inhibitor screens, the substrate concentration is in the range of 10–100 $\mu$M so that $V_w$ will be about $10^4$–$10^5$-fold larger than $V_A$ and thus $I > I_A$ may hold in many cases. In this case, equation **9** can be simplified to give equation **11**.

$$(K_I^{eff})^{Screen} \cong K_I^*[A]_T^{Screen}\left(\frac{I}{I_A}\right)^{Sceen}$$

$$= K_I^*[A]_T^{Screen}\frac{1}{P_I}\left(\frac{V_w}{V_A}\right)^{Screen} \quad \textbf{(11)}$$

The same $K_I^*$ has been used in both equations **10** and **11**, since this dissociation constant is likely to be the same in both the screen and in man. Also, as indicated in these equations, the value of $[A]_T$ is different in the two systems, but this will not influence the search for inhibitors since this will introduce the same difference for all inhibitors. It is clear from these equations that structural modifications to the inhibitor that produce a lower value of $K_I^*$ will also lead to a decrease in $K_I^{eff}$ and such changes will improve the degree of inhibition in both the screen and in man.

What are the consequences of the fact that $I/I_A$ is much larger in the screen than in man? Since in the scooting mode $PLA_2$ are confined to the interface, it is the value of $X_I^*$ and its relationship to $K_I^*$ that will determine the fraction of enzyme that is inhibited (equation **1**). If the screen is conducted with $[A]_T^{screen} = 10^{-5}$ M and $[I]_T^{screen} = 10^{-8}$ M, and if $P_I$ is $10^3$, the value of $X_I^*$ in the screen will be approximately $10^{-5}$. In man, if we use $[I]_T^{man} = 10^{-8}$ M and the same $P_I = 10^3$ as in the screen, then since $[A]_T^{man}$ is about 0.3 M, $X_I^*$ in man will be about $3 \times 10^{-8}$. Thus, it is clear from this calculation that despite the relatively large value of $I/I_A$ in the screen, $X_I^*$ in the screen will be large enough so that the inhibition will not be missed. It is also important to note that an inhibitor that functions well in the screen will likely function equally well or even better in man, since a larger fraction of $I_T$ will be in the membrane phase in man. There is one possible exception to this generalization. In man an inhibitor could be substantially partitioned into interfaces that are not available to the enzyme, i.e. $f_{A*}$ is very small. Under these conditions $K_I^{eff}$ would increase.

The main consequence of the large value of $I/I_A$ in the screen is that $K_I^{eff}$ is a function of both $K_I^*$ and $P_I$ (equation **10**), whereas $K_I^{eff}$ in man is almost always independent of $P_I$ (equation **11**). Thus, if structural changes are made to the inhibitor that change the value of $K_I^{eff}$ in the screen (and thus the degree of inhibition), it will not be immediately obvious whether the increase in potency is due to changes in $P_I$, $K_I^*$, or both. A simple solution to this problem is to re-run the *in vitro* analysis using a five- to ten-fold higher value of $[A]_T^{screen}$. If $K_I^{eff}$ changes in proportion to $[A]_T^{screen}$ (equation **11**), then the change in inhibitor potency is mainly due to a change in $K_I^*$. However, if the effect is due only to a change in $P_I$, then equation **11** applies, and since $[A]_T$ and $V_A$ change by the same factor, the observed $K_I^{eff}$ will not change when $[A]_T$ is increased.

In summary, by using the scooting assay to search for $PLA_2$ inhibitors, water soluble compounds that bind to E and prevent its interfacial binding probably will not be found. However, it is hard to imagine a screen that would find such compounds without the interference from numerous nonspecific agents that modulate the E to E* equilibrium. Furthermore, the scooting analysis yields the value of $K_I^*$ and inhibitors with sufficiently small values of this dissociation constant are guaranteed to function *in vivo*. The only problem that arises in translating the effectiveness of the inhibitor from *in vitro* system to the *in vivo* system is due to partitioning effects; however, this problem is readily remedied as discussed above.

### Epilogue

Interfacial enzymology involves the interplay of physical and interfacial processes with biochemical catalysis. Such enzymes must access the substrate at the interface because the concentration of solitary monomeric substrate molecules in the aqueous phase is very low, and also the rate of substrate desorption from the interface to the aqueous phase is very low. The microinterface between the bound enzyme and the organized substrate not only facilitates formation of the enzyme–substrate complex, but the residence time of the enzyme at the interface controls the extent of processivity at the interface. The minimum kinetic model shown in Fig. 1 permits adaptation of the Michaelis–Menten formalism as a basis to accommodate virtually all aspects of interfacial catalysis. The binding of the enzyme to the interface has two extreme kinetic consequences (Fig. 3): during catalysis in the scooting mode, binding of the enzyme to the interface influences only the pre-steady state portion of the progress curve; on the other hand, catalysis in which the enzyme hops among the ensemble of vesicles, the E to E* step, becomes a part of each catalytic cycle in the steady-state. Complex kinetic consequences with different extents of processivity are predicted[14,26] if the enzyme hops after a few catalytic turnovers. The intervesicle exchange of the enzyme, substrate and products creates further time-dependent changes in what the enzyme 'sees' during the progress of the reaction. In the absence of such parallel rate processes, as is the case in the scooting mode, the kinetics of interfacial catalysis is considerably simplified.[15-20]

The kinetic studies on the characterization of specific competitive inhibitors of $PLA_2$ have also come of age. Kinetic analysis of $PLA_2$ in the scooting mode provides a reliable method for random screening of large compound banks in order to find novel lead compounds. It is also possible to obtain very potent inhibitors and to determine their interfacial $K_I^*$ values by using three independent methods. With the help of the inhibitors available so far, it is now possible to address detailed mechanistic questions about the catalytic steps and the nature of the transition state, and to begin to ask questions related to the biological role of phospholipases $A_2$.

### References

1. Burch RM. G Protein Regulation of Phospholipase $A_2$. *Molecular Neurobiol* 1989: **3**: 155–171.
2. Waite M. *The Phospholipases*. New York: Plenum, 1987.
3. Johnson LK, Frank S, Vadas P, Pruzanski W, Lusis AJ, Seilhamer JJ. Localization and evolution of two human phospholipase $A_2$ genes and two related genetic elements. In: Wong PYK, Dennis EA, eds. *Phospholipase $A_2$: Role and Function in Inflammation*. New York: Plenum Press, 1990; 17–34.
4. Van den Bergh CJ, Slotboom AJ, Verheij HM, de Haas GH. The Role of Asp-49 and Other Conserved Amino Acids in Phospholipase $A_2$ and Their Importance for Enzymatic Activity. *J Cellular Biochemistry* 1989; **39**: 379–390.
5. Verheij HM, Slotboom AJ, de Haas GH. Phospholipase $A_2$: A Model for Membrane-Bound Enzymes? *Rev Physiol Biochem Pharmacol* 1981; **91**: 91–203.
6. Scott DL, White SP, Otwinowski Z, Yuan W, Gelb MH, Sigler PB. Interfacial Catalysis: The Mechanism of Phospholipase $A_2$. *Science* 1990; **250**: 1541–1546.
7. Thunnissen MMGM, Ab E, Kalk KH, *et al.* X-ray Structure of Phospholipase $A_2$ Complexed with a Substrate-Derived Inhibitor. *Nature* 1990; **347**: 689–691.
8. Renetseder R, Brunie S, Dijkstra BW, Drenth J, Sigler PB. A Comparison of the Crystal Structures of Phospholipase $A_2$ from Bovine Pancreas and *Crotalus atrox* Venom. *J Biol Chem* 1985; **260**: 11627–11634.
9. White SP, Scott DL, Otwinowski Z, Gelb MH, Sigler PB. Crystal Structure of Cobra Venom Phospholipase $A_2$ in a Complex with a Transition-State Analogue. *Science* 1990; **250**: 1560–1563.
10. Clark JD, Lin L, Kriz RW, Ramesha CS, Sultzman LA, Lin AY, Milona N, Knopf JL. A Novel Arachidonic Acid-Selective Cytosolic $PLA_2$ Contains a Ca-Dependent Translocation Domain with Homology to PKC and GAP. *Cell* 1991; **65**: 1043–1051.
11. Kramer RM, Roberts EF, Manetta J, Putnam JE. The $Ca^{2+}$-Sensitive Cytosolic Phospholipase $A_2$ is a 100 kDA Protein in Human Monoblast U937 Cells. *J Biol Chem* 1991; **266**: 5268–5272.
12. Leslie CC, Voelker DR, Channon JY, Wall MM, Zelarney PT. Properties and Purification of an Arachidonoyl-Hydrolyzing Phospholipase $A_2$ from a Macrophage Cell Line, RAW 164.7. *Biochim Biophys Acta* 1988; **963**: 476–492.
13. Ghomashchi F, Schuttel S, Jain MK, Gelb MH. Kinetic Analysis of a High Molecular Weight Phospholipase $A_2$ from Rat Kidney: Divalent metal-dependent trapping of enzyme on product-containing vesicles. *Biochemistry* 1992; in press.
14. Jain MK, Berg O. Kinetics of Interfacial Catalysis by Phospholipase $A_2$ and Regulation of Interfacial Activation and Inhibition. *Biochim Biophys Acta* 1989; **1002**: 127–156.
15. Berg OG, Yu B-Z, Rogers J, Jain MK. Interfacial Catalysis by Phospholipase $A_2$: Determination of interfacial rate constants. *Biochemistry* 1991; **30**: 7283–7297.
16. Jain MK, Yu B-Z, Rogers J, Ranadive GN, Berg OG. Interfacial Catalysis by Phospholipase $A_2$: Dissociation constants for calcium, substrate, products, and competitive inhibitors. *Biochemistry* 1991; **30**: 7306–7317.
17. Ghomashchi F, Yu B-Z, Berg OG, Jain MK, Gelb MG. Interfacial Catalysis by Phospholipase $A_2$: Substrate specificity in vesicles. *Biochemistry* 1991; **30**: 7318–7329.
18. Jain MK, Ranadive GN, Yu B-Z, Verheij HM. Interfacial Catalysis by Phospholipase $A_2$: Monomeric enzyme is fully catalytically active. *Biochemistry* 1991; **30**: 7330–7340.
19. Jain MK, Rogers J, Berg OG, Gelb MH. Interfacial Catalysis by Phospholipase $A_2$: Activation by substrate replenishment. *Biochemistry* 1991; **30**: 7340–7348.
20. Jain MK, Tao W, Rogers J, Arenson C, Eibl H, Yu B-Z. Active-Site Directed Specific Competitive Inhibitors of Phospholipase $A_2$: Novel transition state analogs. *Biochemistry* 1991; **30**: 10256–10268.
21. Jain MK. *Introduction to Biomembranes*. New York: John Wiley & Sons, 1988; 423.
22. Segel IH. *Enzyme Kinetics*. New York: John Wiley & Sons, 1975.
23. Verger R, de Haas GH. Interfacial Kinetics of Lipolysis. *Ann Rev Biophys Bioeng* 1976; **5**: 77–117.
24. Cevc G, Marsh D. *Phospholipid Bilayers*. New York: John Wiley & Sons, 1987.
25. Jain MK, Vaz WLC. Dehydration of the Lipid-Protein Microinterface on Binding of Phospholipase $A_2$ to Lipid Bilayers. *Biochim Biophys Acta* 1987; **905**: 1–8.
26. Ramirez F, Jain MK. Phospholipase $A_2$ at the Bilayer Interface. *Proteins* 1991; **9**: 229–239.
27. Dennis EA. Phospholipases. *The Enzymes* 1983; **16**: 307–353.
28. Jain MK, Rogers J, de Haas GH. Kinetics of Binding of Phospholipase $A_2$ to Lipid-Water Interface and Its Relationship to Interfacial Activation. *Biochim Biophys Acta* 1988; **940**: 51–62.
29. Noel JP, Deng T, Hamilton KJ, Tsai M-D. Phospholipase $A_2$ Engineering. 3. Replacement of Lysine-56 by Neutral Residues Improves Catalytic Potency Significantly, Alters Substrate Specificity, and Clarifies the Mechanism of Interfacial Recognition. *J Am Chem Soc* 1990; **112**: 3704–3706.
30. Noel JP, Bingman CA, Deng T, *et al.* Phospholipase $A_2$ Engineering. X-ray Structural and Functional Evidence for the Interaction of Lysine-56 with Substrates. *Biochemistry* 1991; **30**: 11801–11811.
31. Fullington DA, Shoemaker DG, Nichols JW. Characterization of Phospholipid Transfer Between Mixed Phospholipid-Bile Salt Micelles. *Biochemistry* 1990; **29**: 879–886.
32. Jain MK, Rogers J, Jahagirdar DV, Marecek JF, Ramirez F. Kinetics of Interfacial Catalysis by Phospholipase $A_2$ in Intravesicle Scooting Mode, and Heterofusion of Anionic and Zwitterionic Vesicles. *Biochim Biophys Acta* 1986; **860**: 435–447.
33. Jain MK, Maliwal BP, de Haas GH, Slotboom AJ. Anchoring of Phospholipase $A_2$: The effect of anions and deuterated water, and the role of the N-terminus region. *Biochim Biophys Acta* 1986; **860**: 448–461.
34. Jain MK, Rogers J, Marecek JF, Ramirez F, Eibl H. Effect of the Structure of Phospholipid on the Kinetics of Intravesicle Scooting of Phospholipase $A_2$. *Biochim Biophys Acta* 1986; **860**: 462–474.
35. Jain MK, de Haas GH, Marecek JF, Ramirez F. The Affinity of Phospholipase $A_2$ for the Interface of the Substrate and Analogs. *Biochim Biophys Acta* 1986; **860**: 475–483.
36. Jain MK, Egmond MR, Verheij HM, Apitz-Castro RJ, Dijkman R, de Haas GH. Interaction of Phospholipase $A_2$ and Phospholipid Bilayers. *Biochim Biophys Acta* 1982; **688**: 341–348.
37. Jain MK, de Haas GH. Structure of 1-Acyl-lysophosphatidylcholine and Fatty Acid Complex in Bilayers. *Biochim Biophys Acta* 1981; **642**: 203–211.
38. Jain MK, Jahagirdar DV. Action of Phospholipase $A_2$ on Bilayers: Effect of Fatty Acid and Lysophospholipid Additives on the Kinetic Parameters. *Biochim Biophys Acta* 1985; **814**: 313–318.
39. Jain MK, Yu B-Z, Kozubek A. Binding of Phospholipase $A_2$ to Zwitterionic Bilayers Is Promoted by Lateral Segregation of Anionic Amphiphiles. *Biochim Biophys Acta* 1989; **980**: 23–32.
40. Jain MK, Yuan W, Gelb MH. Competitive Inhibition of Phospholipase $A_2$ in Vesicles. *Biochemistry* 1989; **28**: 4135–4139.
41. Jain MK, Jahagirdar DV. Action of Phospholipase $A_2$ on Bilayers: Effect of inhibitors. *Biochim Biophys Acta* 1985; **814**: 319–326.
42. de Haas GH, Dijkman R, Ransac S, Verger R. Competitive Inhibition of Lipolytic Enzymes. IV. Structural Details of Acylamino Phospholipid Analogues Important for the Potent Inhibitory Effects of Pancreatic Phospholipase $A_2$. *Biochim Biophys Acta* 1990; **1046**: 249–257.
43. Radvanyi F, Jordan L, Russo-Marie F, Bon C. A Sensitive and Continuous Fluorometric Assay for Phospholipase $A_2$ Using Pyrene-Labelled Phospholipids in the Presence of Serum Albumin. *Anal Biochem* 1989; **177**: 103–109.
44. Thuren T, Virtanen JA, Lalla M, Kinnunen KJ. Fluorometric Assay for Phospholipase $A_2$ in Serum. *Clinical Chemistry* 1985; **31**: 714–717.
45. Hendrickson HS, Rauk PN. Continuous Fluorometric Assay of Phospholipase $A_2$ with Pyrene Labelled Lecithin as a Substrate. *Anal Biochem* 1981; **116**: 553–558.

46. Elsbach P, Weiss J. Utilization of Labelled *Escherichia coli* as Phospholipase Substrate. *Methods in Enzymology* 1991; **197**: 24–31.
47. Jain MK, Gelb MH. Phospholipase $A_2$ Catalyzed Hydrolysis of Vesicles: Uses of Interfacial Catalysis in the Scooting Mode. *Methods in Enzymology* 1991; **197**: 112–125.
48. Ghomashchi F, Yu B-Z, Mihelich ED, Jain MK, Gelb MH. Kinetic Characterization of Phospholipase $A_2$ Modified by Manoalog. *Biochemistry* 1991; **30**: 9559–9569.
49. Al-Mehdi AB, Dodia C, Jain MK, Fisher AB. Phospholipase $A_2$ ($PLA_2$) Inhibition and Lipid Peroxidation in Lung Ischaemia-Reperfusion. (Submitted).
50. Yuan W, Quinn DM, Sigler PB, Gelb MH. Kinetic and Inhibition Studies of Phospholipase $A_2$ with Short Chain Substrates and Inhibitors. *Biochemistry* 1990; **29**: 6082–6094.
51. Rogers JA, Yu B-Z, Jain MK. Effect of Competitive Inhibitors on the Kinetics of Hydrolysis of Monomeric Substrates by Pig Pancreatic Phospholipase $A_2$. *Biochemistry* 1992; in press.

**Received 18 December 1991; accepted in final form 16 February 1992**

## References

[PDF_01609] Berg O. G., Yu B. Z., Rogers J., Jain M. K. (1991). Interfacial catalysis by phospholipase A2: determination of the interfacial kinetic rate constants.. Biochemistry.

[PDF_01568] Burch R. M. (1989). G protein regulation of phospholipase A2.. Mol Neurobiol.

[PDF_01592] Clark J. D., Lin L. L., Kriz R. W., Ramesha C. S., Sultzman L. A., Lin A. Y., Milona N., Knopf J. L. (1991). A novel arachidonic acid-selective cytosolic PLA2 contains a Ca(2+)-dependent translocation domain with homology to PKC and GAP.. Cell.

[PDF_01695] Elsbach P., Weiss J. (1991). Utilization of labeled Escherichia coli as phospholipase substrate.. Methods Enzymol.

[PDF_01615] Ghomashchi F., Yu B. Z., Berg O., Jain M. K., Gelb M. H. (1991). Interfacial catalysis by phospholipase A2: substrate specificity in vesicles.. Biochemistry.

[PDF_01700] Ghomashchi F., Yu B. Z., Mihelich E. D., Jain M. K., Gelb M. H. (1991). Kinetic characterization of phospholipase A2 modified by manoalogue.. Biochemistry.

[PDF_01690] Hendrickson H. S., Rauk P. N. (1981). Continuous fluorometric assay of phospholipase A2 with pyrene-labeled lecithin as a substrate.. Anal Biochem.

[PDF_01606] Jain M. K., Berg O. G. (1989). The kinetics of interfacial catalysis by phospholipase A2 and regulation of interfacial activation: hopping versus scooting.. Biochim Biophys Acta.

[PDF_01663] Jain M. K., DeHaas G. H., Marecek J. F., Ramirez F. (1986). The affinity of phospholipase A2 for the interface of the substrate and analogs.. Biochim Biophys Acta.

[PDF_01666] Jain M. K., Egmond M. R., Verheij H. M., Apitz-Castro R., Dijkman R., De Haas G. H. (1982). Interaction of phospholipase A2 and phospholipid bilayers.. Biochim Biophys Acta.

[PDF_01697] Jain M. K., Gelb M. H. (1991). Phospholipase A2-catalyzed hydrolysis of vesicles: uses of interfacial catalysis in the scooting mode.. Methods Enzymol.

[PDF_01671] Jain M. K., Jahagirdar D. V. (1985). Action of phospholipase A2 on bilayers. Effect of fatty acid and lysophospholipid additives on the kinetic parameters.. Biochim Biophys Acta.

[PDF_01679] Jain M. K., Jahagirdar D. V. (1985). Action of phospholipase A2 on bilayers. Effect of inhibitors.. Biochim Biophys Acta.

[PDF_01657] Jain M. K., Maliwal B. P., DeHaas G. H., Slotboom A. J. (1986). Anchoring of phospholipase A2: the effect of anions and deuterated water, and the role of N-terminus region.. Biochim Biophys Acta.

[PDF_01618] Jain M. K., Ranadive G., Yu B. Z., Verheij H. M. (1991). Interfacial catalysis by phospholipase A2: monomeric enzyme is fully catalytically active at the bilayer interface.. Biochemistry.

[PDF_01621] Jain M. K., Rogers J., Berg O., Gelb M. H. (1991). Interfacial catalysis by phospholipase A2: activation by substrate replenishment.. Biochemistry.

[PDF_01640] Jain M. K., Rogers J., DeHaas G. H. (1988). Kinetics of binding of phospholipase A2 to lipid/water interfaces and its relationship to interfacial activation.. Biochim Biophys Acta.

[PDF_01653] Jain M. K., Rogers J., Jahagirdar D. V., Marecek J. F., Ramirez F. (1986). Kinetics of interfacial catalysis by phospholipase A2 in intravesicle scooting mode, and heterofusion of anionic and zwitterionic vesicles.. Biochim Biophys Acta.

[PDF_01660] Jain M. K., Rogers J., Marecek J. F., Ramirez F., Eibl H. (1986). Effect of the structure of phospholipid on the kinetics of intravesicle scooting of phospholipase A2.. Biochim Biophys Acta.

[PDF_01624] Jain M. K., Tao W. J., Rogers J., Arenson C., Eibl H., Yu B. Z. (1991). Active-site-directed specific competitive inhibitors of phospholipase A2: novel transition-state analogues.. Biochemistry.

[PDF_01634] Jain M. K., Vaz W. L. (1987). Dehydration of the lipid-protein microinterface on binding of phospholipase A2 to lipid bilayers.. Biochim Biophys Acta.

[PDF_01674] Jain M. K., Yu B. Z., Kozubek A. (1989). Binding of phospholipase A2 to zwitterionic bilayers is promoted by lateral segregation of anionic amphiphiles.. Biochim Biophys Acta.

[PDF_01612] Jain M. K., Yu B. Z., Rogers J., Ranadive G. N., Berg O. G. (1991). Interfacial catalysis by phospholipase A2: dissociation constants for calcium, substrate, products, and competitive inhibitors.. Biochemistry.

[PDF_01677] Jain M. K., Yuan W., Gelb M. H. (1989). Competitive inhibition of phospholipase A2 in vesicles.. Biochemistry.

[PDF_01669] Jain M. K., de Haas G. H. (1981). Structure of 1-acyl lysophosphatidylcholine and fatty acid complex in bilayers.. Biochim Biophys Acta.

[PDF_01571] Johnson L. K., Frank S., Vades P., Pruzanski W., Lusis A. J., Seilhamer J. J. (1990). Localization and evolution of two human phospholipase A2 genes and two related genetic elements.. Adv Exp Med Biol.

[PDF_01596] Kramer R. M., Roberts E. F., Manetta J., Putnam J. E. (1991). The Ca2(+)-sensitive cytosolic phospholipase A2 is a 100-kDa protein in human monoblast U937 cells.. J Biol Chem.

[PDF_01599] Leslie C. C., Voelker D. R., Channon J. Y., Wall M. M., Zelarney P. T. (1988). Properties and purification of an arachidonoyl-hydrolyzing phospholipase A2 from a macrophage cell line, RAW 264.7.. Biochim Biophys Acta.

[PDF_01647] Noel J. P., Bingman C. A., Deng T. L., Dupureur C. M., Hamilton K. J., Jiang R. T., Kwak J. G., Sekharudu C., Sundaralingam M., Tsai M. D. (1991). Phospholipase A2 engineering. X-ray structural and functional evidence for the interaction of lysine-56 with substrates.. Biochemistry.

[PDF_01685] Radvanyi F., Jordan L., Russo-Marie F., Bon C. (1989). A sensitive and continuous fluorometric assay for phospholipase A2 using pyrene-labeled phospholipids in the presence of serum albumin.. Anal Biochem.

[PDF_01637] Ramirez F., Jain M. K. (1991). Phospholipase A2 at the bilayer interface.. Proteins.

[PDF_01586] Renetseder R., Brunie S., Dijkstra B. W., Drenth J., Sigler P. B. (1985). A comparison of the crystal structures of phospholipase A2 from bovine pancreas and Crotalus atrox venom.. J Biol Chem.

[PDF_01580] Scott D. L., White S. P., Otwinowski Z., Yuan W., Gelb M. H., Sigler P. B. (1990). Interfacial catalysis: the mechanism of phospholipase A2.. Science.

[PDF_01688] Thuren T., Virtanen J. A., Lalla M., Kinnunen P. K. (1985). Fluorometric assay for phospholipase A2 in serum.. Clin Chem.

[PDF_01630] Verger R. (1976). Interfacial enzyme kinetics of lipolysis.. Annu Rev Biophys Bioeng.

[PDF_01578] Verheij H. M., Slotboom A. J., de Haas G. H. (1981). Structure and function of phospholipase A2.. Rev Physiol Biochem Pharmacol.

[PDF_01589] White S. P., Scott D. L., Otwinowski Z., Gelb M. H., Sigler P. B. (1990). Crystal structure of cobra-venom phospholipase A2 in a complex with a transition-state analogue.. Science.

[PDF_01703] Yuan W., Quinn D. M., Sigler P. B., Gelb M. H. (1990). Kinetic and inhibition studies of phospholipase A2 with short-chain substrates and inhibitors.. Biochemistry.

[PDF_01681] de Haas G. H., Dijkman R., Ransac S., Verger R. (1990). Competitive inhibition of lipolytic enzymes. IV. Structural details of acylamino phospholipid analogues important for the potent inhibitory effects on pancreatic phospholipase A2.. Biochim Biophys Acta.

[PDF_01575] van den Bergh C. J., Slotboom A. J., Verheij H. M., de Haas G. H. (1989). The role of Asp-49 and other conserved amino acids in phospholipases A2 and their importance for enzymatic activity.. J Cell Biochem.

